# External stress, formaldehyde, and schizophrenia: a new mouse model for mental illness research

**DOI:** 10.1038/s41537-025-00603-3

**Published:** 2025-03-26

**Authors:** Junhao Cheng, Zihui Sun, Hao Zhang, Danrui Zhao, Panpan Wang, Haishu Chen, Wanjia Lyv, Qiangfeng Deng, Yuanyu Fu, Xingzhou Lyv, Tingting Gao, Jinan Xu, Feiyan Zhou, Yiqing Wu, Xu Yang, Ping Ma, Zhiqian Tong

**Affiliations:** 1 Zhejiang Provincial Clinical Research Center for Mental Disorders, The Affiliated Wenzhou Kangning Hospital, School of Mental Health, Wenzhou Medical University, Wenzhou, Zhejiang 325035 P.R. China; 2 Wenzhou semir united international school, Wenzhou, China; 3 Beijing Geriatric Hospital, Beijing, 100049 China; 4 Key Laboratory of Environmental Related Diseases and One Health, Xianning Medical College, Hubei University of Science and Technology, Xianning, 437100 China; 5 University of Alberta, Edmonton, AB Canada

**Keywords:** Schizophrenia, Human behaviour

## Abstract

Although MK801-induced NMDA receptor (NMDAR) hypofunction mimics schizophrenia symptoms, the exact factors causing NMDAR inhibition are unknown. Unexpectedly, external stress elicits formaldehyde (FA) generation; FA can induce depression and cognitive impairments by blocking NMDARs. This study explores using FA injection to establish a schizophrenia-like model in mice. Here, we reported that external stress-derived FA induces schizophrenia-like behaviors. Four experimental methods were used to induce schizophrenia-like symptoms in wild-type mice: double electrode stimulation of the ventral tegmental area (VTA), microinjection of FA or tetrahydroisoquinoline (TIQ) into the VTA, and intraperitoneal injection of MK801. Then the metabolic levels of FA and dopamine (DA) in the prefrontal cortex (PFC) and VTA were quantified using ELISA kits. We found that external stress-electrical stimulation via VTA caused schizophrenia-like behaviors, including despairing behavior as measured by the tail suspension test, anhedonia as evaluated by the sucrose preference test, stereotypical behavior as assessed by the marble burying test (MBT), anxiety-like behavior as measured by the open-field test and memory deficit as detected by the Y-maze. These behaviors correlated with increased DA and TIQ levels in the VTA and decreased DA levels in the PFC. High-resolution mass spectrometry (HRMS) and high-performance liquid chromatography (HPLC) confirmed TIQ formation from FA and DA. Furthermore, injecting TIQ into the VTA induced schizophrenia-like symptoms in mice, marked by higher FA and lower DA levels in the PFC than control mice. Strikingly, injecting FA into the VTA as well as administering MK-801 induced schizophrenia-like behaviors associated with reduced DA levels and low activity of tyrosine hydroxylase (TH) and monoamine oxidase (MAO) in the PFC. Hence, microinfusion of FA into the VTA can be used to prepare schizophrenia-like changes mouse model, suggesting that stress-derived FA may act as an endogenous trigger of schizophrenia-like changes.

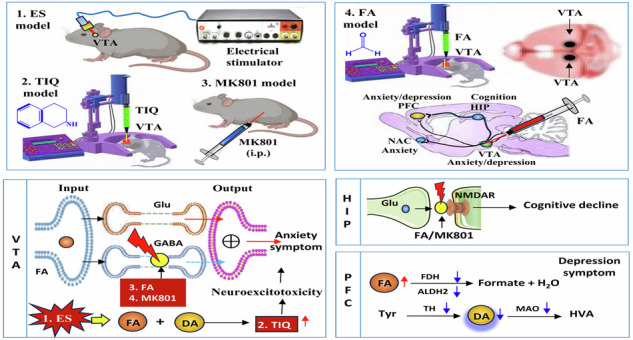

## Introduction

Schizophrenia is acommon group of severe mental disorders of undetermined etiology, with a prevalence of up to 1% of the world’s population^1,2^. The World Health Organization states in its latest report for 2022 that around 24 million people worldwide suffer from schizophrenia^3^. It is characterized by high persistence, high recurrence and high disability, and the average life expectancy of patients is 15–20 years shorter than that of the general population^4,5^. Schizophrenia involves interactions between multiple genes and a range of environmental and psychosocial factors may also influence the onset and course of this disease^6^. The unclear pathogenesis of schizophrenia complicates diagnosis and treatment, often leading to relapse. Animal models can offer insights into schizophrenia-related behaviors that overlap with other psychiatric disorders, aiding in understanding the disease and developing new drugs^7,8^.

Schizophrenia is a major psychotic disorder characterized by positive symptoms (hallucinations and delusions), negative symptoms (avolition and anhedonia), anxiety, depression, and cognitive dysfunction^9–11^. Many hypotheses, such as the genetic and environmental factors, neurotransmitter disorders, etc. have been proposed^12–14^; however, one of the most recognized hypotheses is the dopamine (DA) hypothesis, in which excessive dopaminergic neurotransmission in the midbrain limbic leads to positive symptoms, and low dopamine activity in the midbrain cortex is associated with negative and cognitive symptoms^9,15^. N-methyl-D-aspartate (NMDA) receptor (NMDAR) hypofunction contributes to the pathophysiology of schizophrenia^16^. It has also been found in autism, Alzheimer’s disease and cognitive dementia^17^. NMDAR antagonist models and the anti-NMDAR autoantibody model of schizophrenia support the viewpoint that NMDAR hypofunction could occur in GABAergic neurons in both models^18,19^. However, which exogenous or endogenous factor causing NMDAR inhibition are unknown.

In 1980, hyperformaldehydism was proposed to contribute to schizophrenia onset^20^. Recently, exogenous FA has been found to induce anxiety- and depressive-like behaviors^21,22^, and cognitive impairments by inhibiting NMDAR function^23,24^. Notably, external stimulations including electrical stimulation (ES) and spatial training can induce a transient generation of endogenous FA in the brain^23^. Especially, excess FA can react with DA to produce tetrahydroisoquinoline (TIQ) in the substantia nigra and striatum^25^, which results in schizophrenia-like symptoms in animals^26,27^. Electroshock to the dopaminergic neuron-rich midbrain (VTA) leads to schizophrenic-like behavior in rats^28^. These data strongly suggest that FA-blocked NMDAR and FA-related TIQ formation contribute to schizophrenia-like changes onset.

In this study, we sought to develop an innovative murine model of schizophrenia-like changes through the microinjection of FA into the VTA. Four experimental methods were used to induce schizophrenia-like behaviors in wild-type mice: double electrode stimulation of VTA^29^, microinjection of FA or TIQ into the VTA, and intraperitoneal injection of MK801^30,31^. Then the metabolic levels of FA and DA in the PFC and VTA were measured using ELISA kits. Our findings indicate that the administration of FA into the VTA elicits the elevated activity in VTA-associated positive behaviors while concurrently resulting in the diminished activity in PFC-associated negative behaviors in mice, thereby replicating the symptomatic manifestations observed in clinical schizophrenia-like changes (Graphic abstract).

## Materials and methods

### Animals

In this experiment, 8–9-week-old specific pathogen free C57BL/6J male mice were utilized, all of which were procured from Beijing Vetonglihua Laboratory Animal Technology Co., Ltd. The experimental animal license number was SYXK (Zhe) 2021-0020. The mice were housed in cages with a density of 4–5 mice per cage. All mice were maintained in individually ventilated cage (IVC) barrier systems under controlled conditions, including a constant temperature of 23 ± 2 °C, humidity levels of 40–60%, and a 12-h light/dark cycle (8:30–20:30). The experiments and experimental protocols were approved by The Medical Ethics Committee of Wenzhou Medical University (wydw2022-0545). Throughout the experiments, measures were implemented to minimize animal suffering. All surgical procedures and euthanasia were conducted utilizing 1.25% 2,2,2-tribromoethanol at a dosage of 0.2 mL per 10 g of body weight, then these mice were removed from the jar and cervical dislocation is performed to assure euthanasia.

### Electrical stimulation of VTA model

#### Pre-operative preparation

All surgical instruments and materials were sterilized using high-pressure methods. The coordinates of the VTA region were determined using mouse brain stereotactic atlas as follows: anteroposterior (AP): −3.3 mm; mediolateral (ML): ±0.5 mm; dorsoventral (DV): −4.4 mm. Bilateral electrodes were implanted at these coordinates (AP: −3.3 mm; ML: ±0.5 mm; DV: −4.4 mm)^29^. Anesthesia was induced in the mouse using 1.25% 2,2,2-tribromoethanol at a dosage of 0.2 mL per 10 g of body weight.

#### Surgery

Upon confirming complete anesthesia, the mouse’s incisors were positioned within the incisal bars, and the head was stabilized and aligned using bilateral ear bars. The surgical site was then shaved and disinfected with an iodophor wipe. An incision was made along the median sagittal axis of the head’s skin. Subsequently, cranial holes were meticulously drilled vertically with a dental drill.

#### Buried electrodes

Customized electrodes according to VTA coordinates (AP: −3.3 mm; ML: ±0.5 mm; DV: −4.4 mm) (KDO Brain Machine Technology Co., Ltd.). The procedures for preoperative preparation, surgery, and brain stereotactic injection were consistent across all subjects. Shallow holes were drilled at designated locations, and cranial nails were inserted. Electrodes were clamped and gradually inserted into the target brain region using a stereotactic device. Both the cranial nails and electrodes were then secured with dental cement. Following the setting of the dental cement, the electrodes were carefully released. The mice were subsequently placed on a heating pad to facilitate recovery before being returned to their original cages.

#### ES model

The electrodes were implanted in the VTA of the mice, which were allowed a recovery period of two weeks. Subsequently, the mice underwent electrical stimulation while in a non-stressed condition. The electrostimulator was purchased from Shanghai Yuyan Scientific Instrument Co., Ltd., model: 9A; the stimulation parameters were: wave width 1 ms, frequency 100 Hz, duration 2 s, current 50 μA, stimulation twice, stimulation interval 5 min^29^.

### Microinjection of TIQ into VTA by stereotactic injection

TIQ (#T13005, Merck, USA) was dissolved in saline and its pH was adjusted to 4, the optimal solubility pH for TIQ, using a phosphate buffer pair. The TIQ solution was then diluted to a concentration of 20 mM with volume of 2 μL for subsequent injection into the VTA of the experimental group. Concurrently, saline was similarly adjusted to pH 4 with the phosphate buffer pair and used for injections into the VTA of the control group. All procedural steps, with the exception of the rest period, adhered to the established FA brain stereotactic injection protocol.

### Microinfusion of FA into VTA by stereotactic injection

A volume of 2 μL of PBS was injected into the VTA region of the control mouse at a rate of 0.2 μL/min using a 10 μL Hamilton syringe controlled by a micropump; 10 mM FA (Formalin, #65346-M, 10%, Merck, USA) in PBS was injected into the VTA region of the modeling group of mice in the same volume. After injection, the needle was left at the original injection site for 5 min to wait for the solution to diffuse. After injection, withdraw the syringe slowly to prevent leakage of the solution. The wound was closed briefly and interrupted using absorbable sutures and sterilized with iodophor. Mice were then placed on a heating pad to recover and were returned to their original cage box.

### Intraperitoneal injection of dizocilpine (MK801)

The mice were modeled by intraperitoneal injection of dizocilpine (MK801, Maclean’s, #M794425-1ml, USA) at a dose of 0.5 mg/kg 30 min before the start of the experiment as previously reported^30,31^.

### Behavioral assessments in mice

#### Marble-burying test

Two days before training, two glass marbles were placed in the mouse cage to prevent object phobia during testing. On test day, 5 cm thick bedding was added to a similarly sized cage. Twelve marbles were evenly distributed in the test cage. Mice were gently placed in the cage and allowed to move freely for 15 min. After the test, the mice were removed, and the number of marbles buried at least two-thirds was recorded and calculated as a percentage.

#### Open field test

Using an open field (#63008, Reward, China), a 30 $*$ 30 cm area in the middle of the bottom surface was recorded as previous report^22^.

#### Tail suspension test

Timing was started by using a suspension tail box (#JLBehv-STGM, YUYAN INSTRUMENTS, China) medical tape to secure the tip of the mouse’s tail at 1–2 cm, suspended from the box as previous report^22^. A video recording, enabled by detection software, is employed to document the behavioral responses. The experimental duration is established at six minutes, during which the hanging time of the mouse is recorded. The period of immobility is assessed during the concluding four minutes, defined by the cessation of struggle or absence of activity in the mice.

#### Sugar preference test

The experiment lasted for a total of three days, including 24 h of acclimatization, 24 h of fasting and water restriction, and 24 h of formal testing. Each mouse should be kept in a single cage for 24 h for acclimatization and be carried out for behavior test as previous report^22^. The mice were provided with two bottles of water, one containing 150 mL of a 1% sucrose solution and the other containing 150 mL of plain water, for a duration of 24 h. Sugar water preference index = Sugar water consumption/total liquid consumption * 100%.

#### Y-maze

A Y-maze (#63006, Reward, China) constructed from perspex was installed on an opaque square perspex board (64.5 cm × 56.5 cm). The shield of the Y-maze was 20 cm high and 0.5 cm thick. Each arm was 30 cm long and 8 cm wide. The maze was placed in a room containing lots of extra-maze cues^32^. The mice are gently positioned at the maze’s center to explore freely for 8 min. Entry into an arm required all four limbs inside. The sequence and total entries into each arm were recorded. A correct spontaneous alternation behavior was recorded when mice entered different arms three times consecutively. Between each experiment, clean the maze with 75% alcohol and let it fully evaporate to prevent odor interference. The spontaneous alternation rate (%) = [(number of spontaneous alternations)/(total number of arm intakes − 2)] × 100.

### Sample preparation

Performed the following steps on ice: Took the centrifuge tube with the target brain region and added pre-cooled PBS (4 °C) at a 1:9 weight ratio. Added steel beads and grind for 60 s at 70 Hz using a tissue grinder. Pre-cooled the centrifuge tube at 4 °C, then centrifuged at 12,000 r/min for 30 min at 4 °C. Carefully aspirated the supernatant, which is the tissue sample. To minimize freeze-thaw cycles, dispensed appropriate amounts based on experimental needs.

### Fluorescence quantification of FA

Na-FA storage solution and working solution were prepared as described previously^23^. Added 7.44 μL of 37% FA solution to 997 μL PBS to make a 100 mM FA solution. Then, mixed 5 μL of this 100 mM FA solution with 445 μL PBS to create a 1 mM FA solution. Prepared the FA standard working solution through gradient dilution as needed. In a black 96-well plate, add 120 μL PBS, 40 μL mouse brain tissue homogenate supernatant or blood, and the FA standard working solution. Added 40 μL Na-FA working solution and incubate for 30 min at room temperature. Measured fluorescence intensity using a multifunctional enzyme marker with an excitation wavelength of 440 nm and an emission wavelength of 543 nm.

### Enzyme-linked immunoassay (ELISA) kits

The different ELISA kits (DA: BLL110130E, BAILILAI BIOLOGY, China; MAO: BLL109103E, BAILILAI BIOLOGY, China; TH: BLL110904E, BAILILAI BIOLOGY, China; ALDH2: BLL109067E, BAILILAI BIOLOGY, China; FDH: BLL106327E, BAILILAI BIOLOGY, China; SSAO: ELL109339E, BAILILAI BIOLOGY, China; SARDH: BLL105453E, BAILILAI BIOLOGY, China) were carried out according to the instructions provided by the manufacturer.

### Co-incubation of dopamine with FA

Prepared the detection mixture as for high-resolution mass spectrometry, but used double-distilled deionized water as the solvent. Diluted the mixture to match the concentration range of the ELISA kit and FA fluorescence quantification assay before detection.

### TIQ identified by high-resolution mass spectrometry (HRMS)

Dissolved 0.0015 g DA in 1 mL of mass spectrometry grade ethanol to make a 10 mM DA solution. Vortex, then diluted 50 μL of this solution with 950 μL ethanol to get a 0.5 mM DA solution. Prepared a 50 mM FA solution by mixing 3.72 μL of 37% FA with 992 μL of double-distilled water. Added 10 μL of the 50 mM FA solution to the 0.5 mM DA solution, vortex to mix, resulting in a 0.5 mM DA and 0.5 mM FA mixed solution. The mixture was incubated at 37 °C for 24 h, then diluted to match the mass spectrometer’s concentration range for positron mass spectrometry analysis^33^.

### TIQ examined by high-performance liquid chromatography (HPLC)

The FA-DA adducts including TIQ need to be cleaned up before being applied to the HPLC as previously reported^34^. A selective pre-purification process utilizing alumina was conducted by transferring the FA samples, which had reacted with DA, into Eppendorf vials containing 10 mg of pre-washed alumina beads. The alumina was prewashed with 1 N HCl and water, then suspended in 500 μL of 0.2 M Tris buffer (pH 8.2) along with 200 μL samples containing isoproterenol as an internal standard. The mixtures were shaken for 10 minutes in the dark at room temperature. The samples were centrifuged for 2 min at 10,000*g*, and the supernatants were discarded. After washing with 200 μL of water, the bound dopamine and FA-DA adducts were eluted from the alumina using HPLC elution buffer (pH 2.75). Aliquots of 50 μL of the eluted supernatant were then analyzed by HPLC^34^.

### Statistical analysis

Data from in vitro experiments were expressed as mean ± standard deviation (SD) and data from in vivo experiments were expressed as mean ± standard error of the mean (SEM). When the experimental data conform to a normal distribution, statistical analysis was performed using t-test or ANOVA. All statistical analyses were performed using GraphPad Prism software. *p* < 0.05 was statistically significant, *p < 0.05, **p < 0.01.

## Results

### Elevated VTA DA levels and reduced PFC DA levels in the ES-model mice

**Fig. 1:**
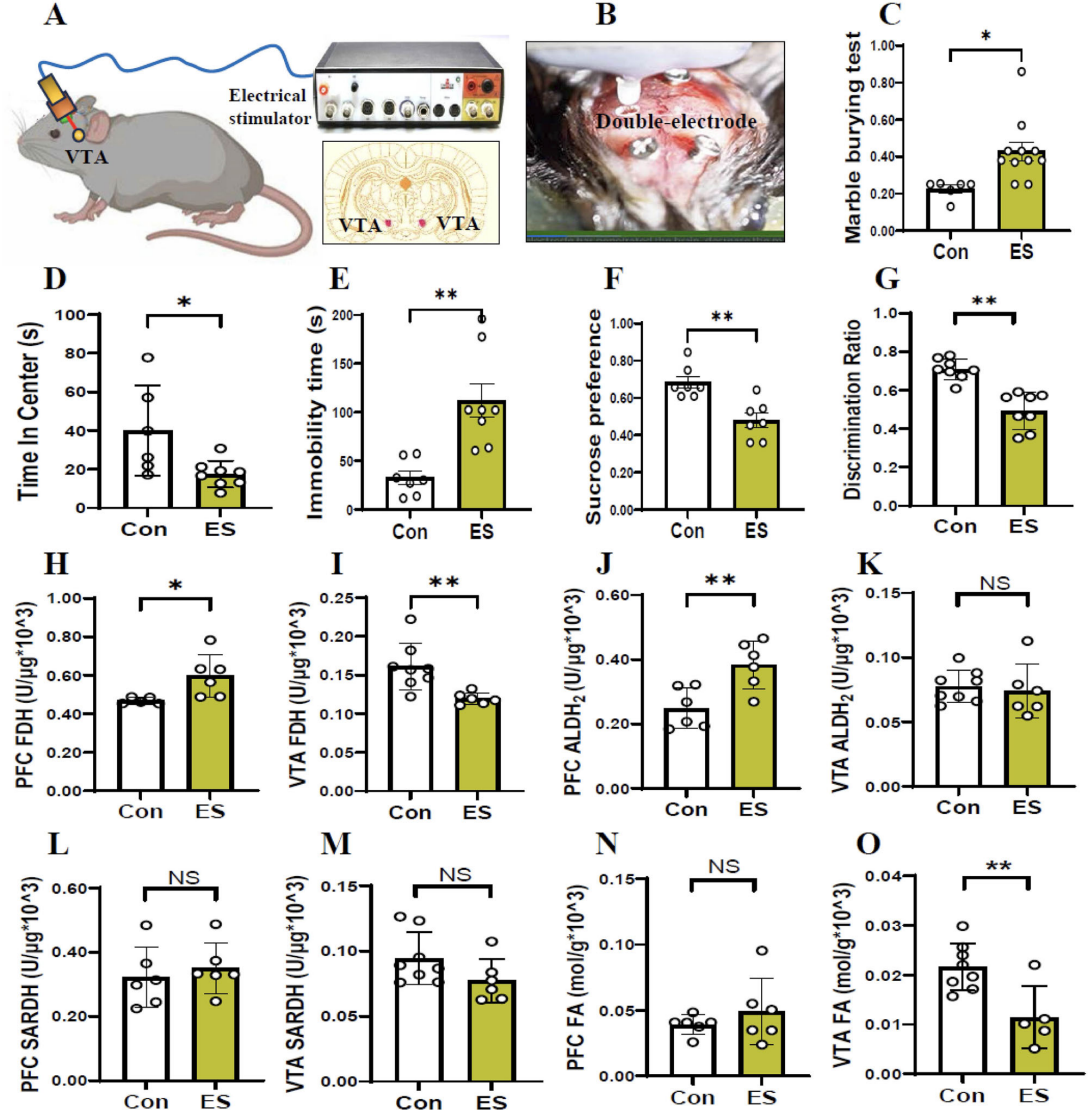
Changes in the depression-like behaviors and the status of FA metabolism in the electrical stimulation-model mice. **A**, **B** Location of double-electrode in the VTA of mice. VTA ventral tegmental area. **C**, **D** Anxiety-like behaviors assessed by the marble burying test and open-field test, respectively. **E**, **F** Depression-lie behaviors evaluated by the tail suspension test and sucrose preference test, respectively. **G** Short-term memory assessed by Y-maze test. **H**, **I** FDH activity in the PFC and VTA detected by ELISA kits. FDH formaldehyde dehydrogenase, PFC prefrontal cortex, VTA ventral tegmental area, ELISA enzyme-linked immunosorbent assay. **J**, **K** ALDH2 activity in the PFC and VTA detected by ELISA kits. **L**, **M** SARDH activity in the PFC and VTA detected by ELISA kits. SARDH sarcosine dehydrogenase. **N**, **O** FA levels in the PFC and VTA detected by NaFA probe. FA: formaldehyde. *p < 0.05; **p < 0.01; NS no statistical significance.

The mouse was electrically stimulated 2 weeks after embedding the electrodes, anxiety-like behaviors in mice were evaluated using the marble buried test (MBT) and the open-field test (OFT). The depression-like symptoms were assessed using tail suspension test (TST) and sucrose preference test (SPT). The short-term memory behaviors were estimated using the Y maze test (YMT). Brain stereotactic localization of VTA and the buried double conductive poles were showed in the Fig. 1A. The VTA region of mice were stimulated by electrical stimulator (Fig. 1B).

Compared with control, the ES-mice showed anxiety-like behaviors, because they had a significant increase in the MBT but a decrease in the movement time in the central zone in the OFT (*t* = 2.887, *p* = 0.011, Fig. 1C; *t* = 2.589, *p* = 0.024, Fig. 1D). These ES-mice also exhibited depression-like behaviors, characterized by an increase in the immobility time in TST and a reduced preference for sugar water compared to the control group (*t* = 4.226, *p* = 0.002, Fig. 1E; *t* = 4.067, *p* = 0.002, Fig. 1F). Notably, the ES-mice showed memory decline than control, because they had lower percentage of alternating behaviors in the YMT than control (*t* = 5.583, *p* < 0.0001, Fig. 1G).

Since FA can inactivate DA^25^, and high levels of DA in the midbrain limbic results in positive symptoms (aggressive behaviors) and anxiety; while low levels of DA in the midbrain cortex is associated with negative and cognitive symptoms^9,15^, we detected the metabolic levels of FA and DA in the VTA and PFC in the ES-mice and control. The results showed that FDH (a FA-degrading enzyme) activity was elevated in the PFC but declined in the VTA in the ES-mice than control (*t* = 2.581, *p* = 0.030, Fig. 1H; *t* = 3.280, *p* = 0.007, Fig. 1I). The activity of ALDH2 (a FA-degrading enzyme) was increased in the PFC but not in the VTA (*t* = 3.414, *p* = 0.007, Fig. 1J; *t* = 0.372, *p* = 0.316, Fig. 1K). In contrast, the activities of SARDH (a FA-generating enzyme) and semicarbazide-sensitive amine oxidase (SSAO, a FA-generating enzyme) in the VTA and PFC in each group were not changed (*t* = 0.566, *p* = 0.584, Fig. 1L; *t* = 1.669, *p* = 0.121, Fig. 1M; *t* = 1.094, *p* = 0.300, Fig. S1A; *t* = 1.640, *p* = 0.130, Fig. S1B). Strikingly, we found that there was a decrease in FA levels in the VTA but not in the PFC in the ES-mice than control (*t* = 0.934, *p* = 0.372, Fig. 1N; *t* = 3.311, *p* = 0.007, Fig. 1O).

**Fig. 2:**
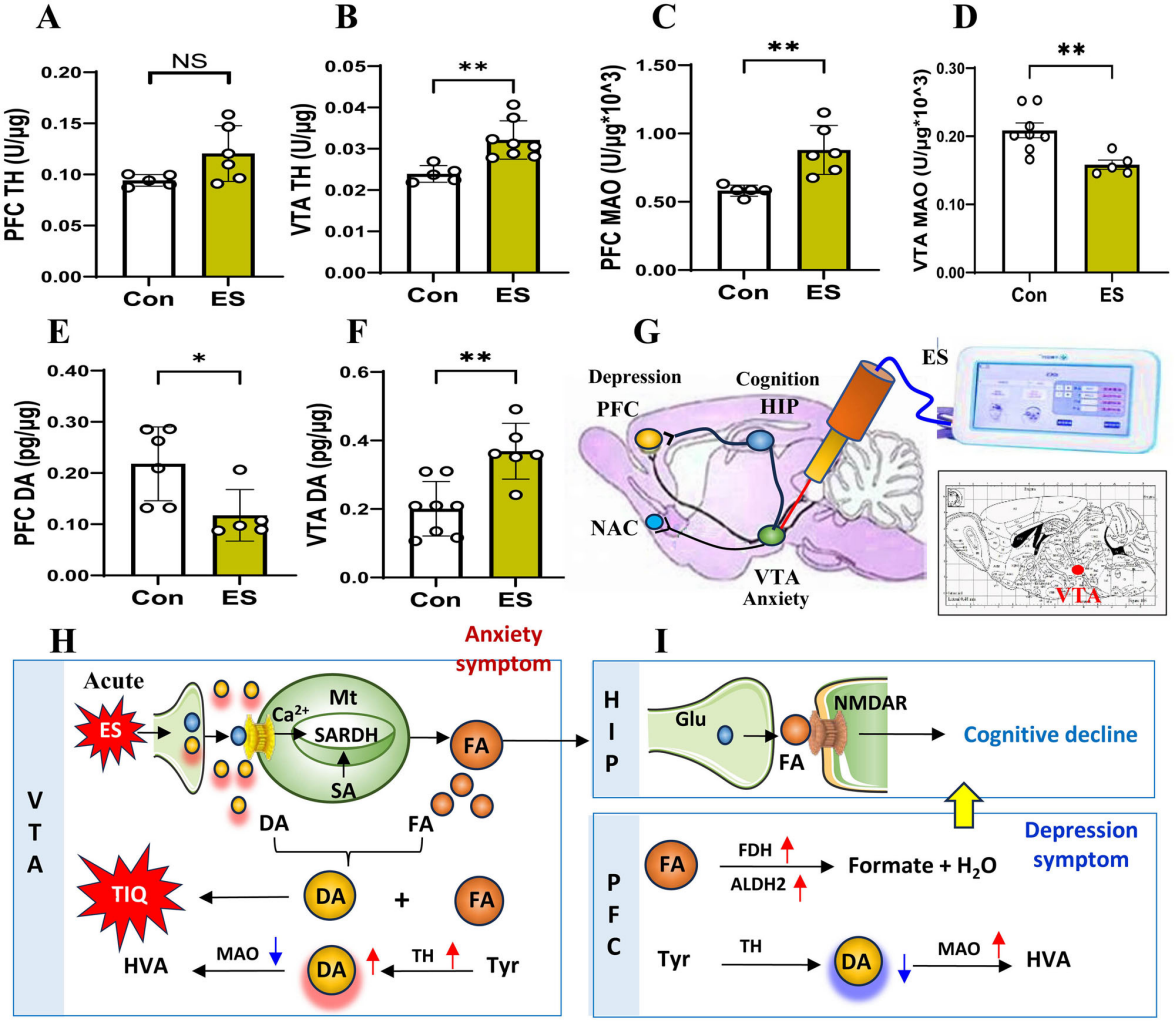
Changes in the metabolic levels of DA in the electrical stimulation-model mice. **A**, **B** TH activity in the PFC and VTA detected by ELISA kits. TH tyrosine dehydrogenase, PFC prefrontal cortex, VTA ventral tegmental area, ELISA enzyme-linked immunosorbent assay. **C**, **D** MAO activity in the PFC and VTA detected by ELISA kits. MAO: monoamine oxygenase. **E**, **F** DA levels in the PFC and VTA quantified by ELISA kits. DA dopamine. **G** Location and stimulation by electrical stimulation. ES electrical stimulation. **H** Anxiety associated with high levels of DA in the VTA. **I** Cognitive decline by FA-blocked NMDAR and depression accompanied by low levels of DA in the PFC. *p < 0.05; **p < 0.01; NS no statistical significance.

Then we examined the metabolic levels of DA in the VTA and PFC in the mice. The results showed that the activity of tyrosine hydroxylase (TH, a rate-limiting enzyme in the DA biosynthesis) was markedly increased in the VTA but not in the PFC in the ES-mice than control (*t* = 2.101, *p* = 0.065, Fig. 2A; *t* = 3.695, *p* = 0.004, Fig. 2B). Moreover, the activity of monoamine oxidase (MAO, a DA-degrading enzyme) was increased in the PFC but decreased in the VTA (*t* = 3.608, *p* = 0.006, Fig. 2C; *t* = 3.374, *p* = 0.006, Fig. 2D). Compared with control, the DA levels were decreased in the PFC while increased in the VTA in the ES-mice (*t* = 2.616, *p* = 0.028, Fig. 2E; *t* = 3.871, *p* = 0.002, Fig. 2F). Above data indicate that acute ES-stimulated VTA leads to FA generation and elicits DA release in the VTA, which may contribute to anxiety onset (Fig. 2G, H). However, FA can be rapidly degraded and had reaction with DA; these low levels of DA in the PFC directly lead to cognitive decline and depression occurrence (Fig. 2I).

### TIQ formation between FA and DA identified by HRMS and HPLC

**Fig. 3:**
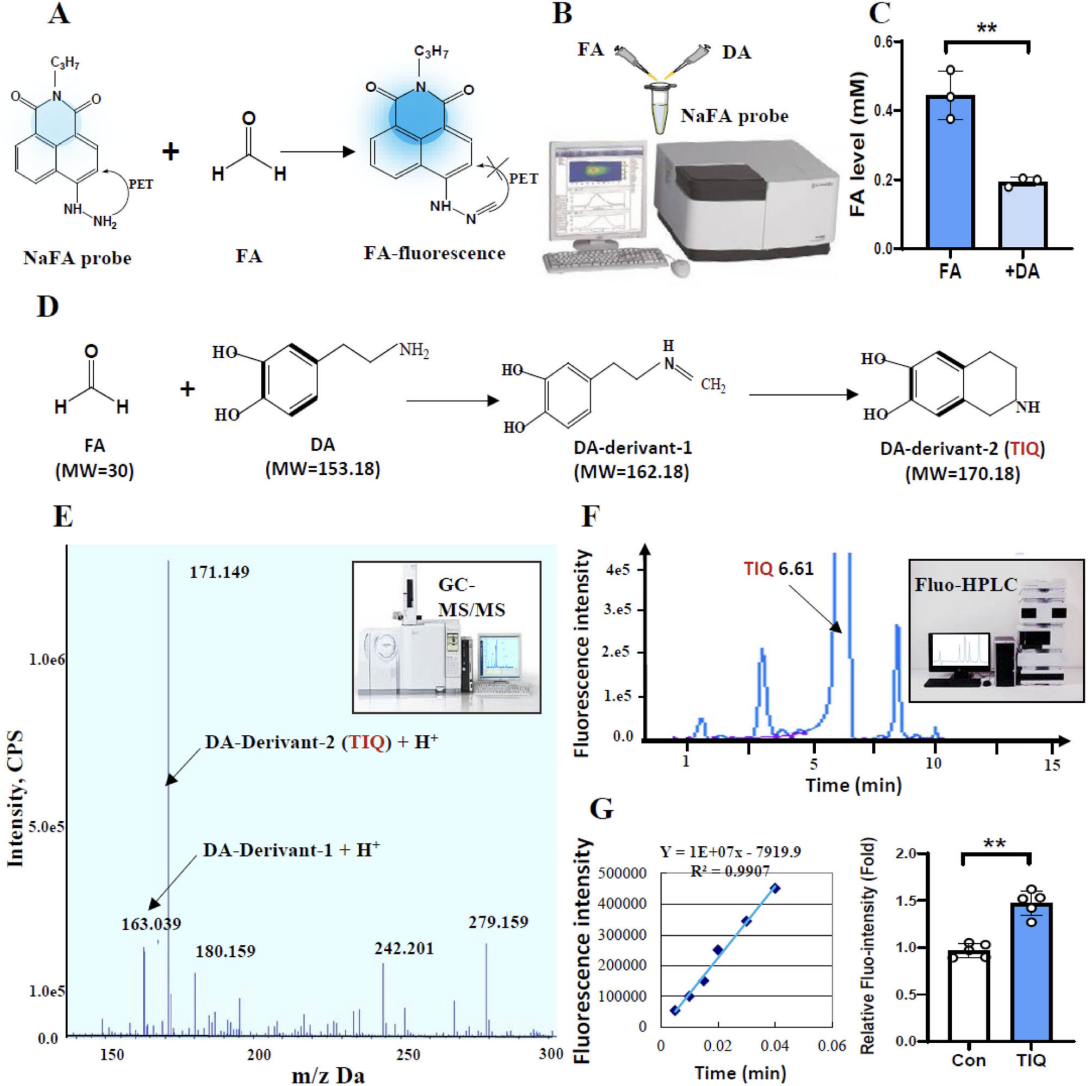
Identification of TIQ in vitro by HRMS and in vivo by HPLC. **A** FA probe- NaFA. **B**, **C** Change in FA levels detected by NaFA probe, FA formaldehyde, DA dopamine. **D** TIQ formation between FA and DA. TIQ tetrahydroisoquinoline. **E** TIQ identified by HRMS. HRMS high-resolution mass spectrometry. **F** TIQ in the brain quantified by HPLC. HPLC high-performance liquid chromatography. **G** Changes in TIQ levels in the ES-model mice. ES electrical stimulation. **p < 0.01.

FA has been proposed to have chemical react with DA to form TIQ^25^, we identified whether TIQ exists in the mixed solutions of DA and FA; especially, in the brain of ES-model mice. First, we used a specific FA-fluorescence probe- (NaFA^23^) to detect the changes in FA concentrations in the mixed solutions of DA and FA (1:1, 37 °C) after co-incubation for 24 h (Fig. 3A). The results showed that FA levels were markedly decline after DA was added into FA solution (Fig. 3B, C).

Theoretically, TIQ can be formed from DA and FA (Fig. 3D)^25^. We used the method of HRMS to detect TIQ in the mixed solution of DA and FA. As expected, TIQ with MW = 170.14 could be identified (Fig. 3E). Using HPLC to quantify the concentrations of TIQ in the brain of mice as previously reported^34^ (Fig. 3F), we found that TIQ levels in the VTA of ES-model mice were markedly higher than that of control (Fig. 3G). These data confirm that FA can inactivate DA to form TIQ.

### Elevated VTA FA levels and reduced PFC DA levels in the TIQ-model mice

**Fig. 4:**
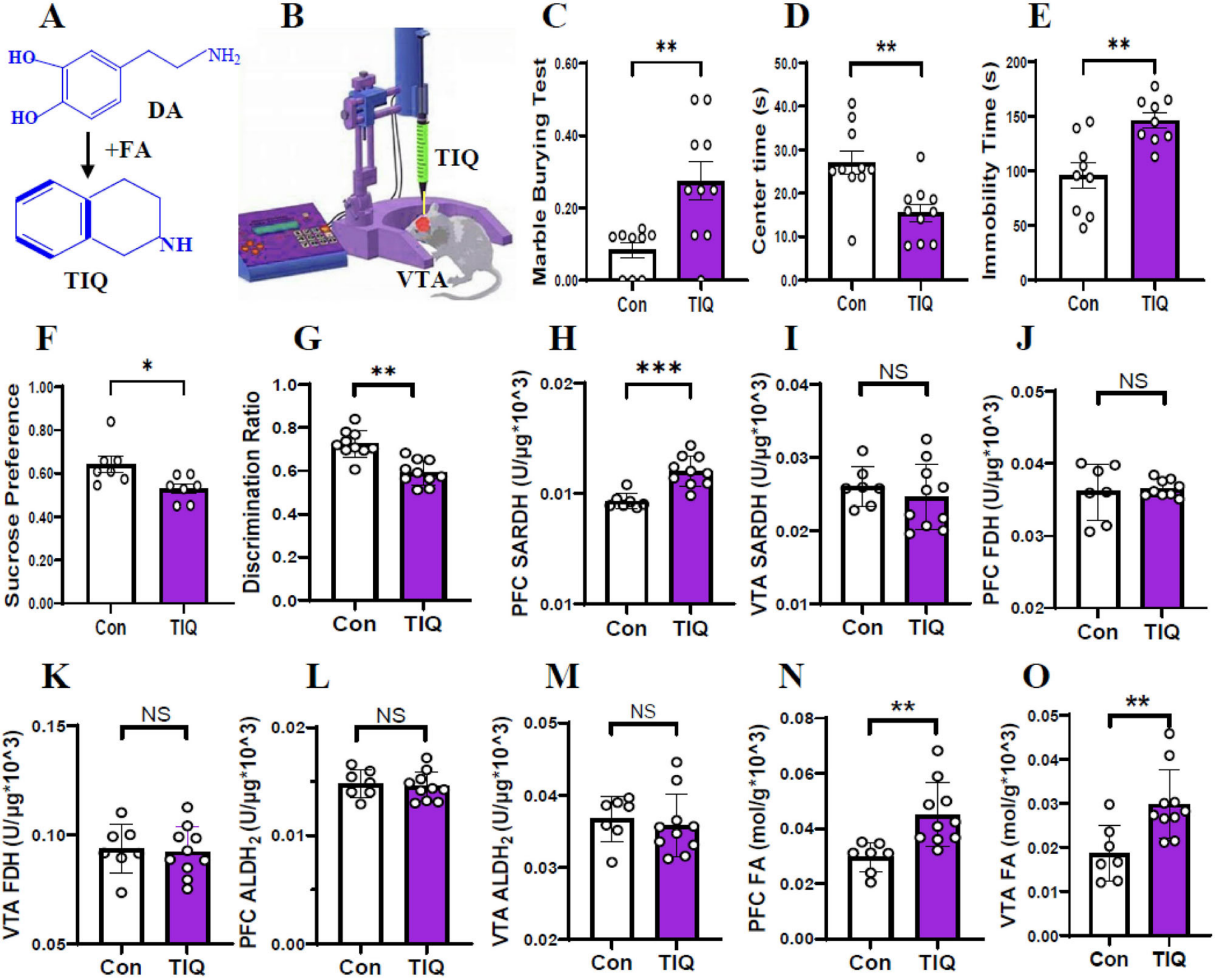
Changes in behaviors and the metabolic levels of FA in the TIQ-model mice. **A**, **B** Microinfusion of TIQ into the VTA in mice. TIQ tetrahydroisoquinoline, VTA ventral tegmental area. **C**, **D** Anxiety-like behaviors assessed by the marble burying test and open-field test, respectively. **E**, **F** Depression-lie behaviors evaluated by the tail suspension test and sucrose preference test, respectively. **G** Short-term memory assessed by Y-maze test. **H**, **I** SARDH activity in the PFC and VTA detected by ELISA kits. SARDH sarcosine dehydrogenase. **J**, **K** FDH activity in the PFC and VTA detected by ELISA kits. FDH formaldehyde dehydrogenase, PFC prefrontal cortex, ELISA enzyme-linked immunosorbent assay. **L**, **M** ALDH2 activity in the PFC and VTA detected by ELISA kits. **N**, **O** FA levels in the PFC and VTA detected by NaFA probe. FA: formaldehyde. *p < 0.05; **p < 0.01; NS no statistical significance.

Previous studies have revealed that TIQ at high concentration has neurotoxicity to dopaminergic neurons^35,36^, and leads to Parkinsonism-like behaviors in mice^37^. Hence, we investigated the effects of excessive TIQ in the VTA on behaviors in mice. Brain stereotactic injection of TIQ (a major product of DA and FA) was applied into VTA of mice (Fig. 4A, B), and then behaviors including anxiety, depression and memory were elevated. We found that the TIQ-mice showed anxiety-like behaviors, because they had an increase in the MBT but a decrease in the movement time in the central zone in the OFT when compared with control (*t* = 3.280, *p* = 0.004, Fig. 4C; *t* = 3.354, *p* = 0.004, Fig. 4D). The TIQ-mice also exhibited depression-like behaviors associated with an increase in the immobility time in TST but a decrease in the preference in the SPT than control (*t* = 3.759, *p* = 0.002, Fig. 4E; *t* = 2.611, *p* = 0.023, Fig. 4F). Strikingly, the TIQ-mice had lower percentage of alternating behaviors in the YMT than control (*t* = 4.995, *p* < 0.0001, Fig. 4G).

To examine the metabolic levels of FA in the VTA and PFC in the TIQ-mice, NaFA probe and ELISA kits were used in this study. The results showed that SARDH activity was elevated in the PFC but not changed in the VTA in the TIQ-mice than control (*t* = 4.774, *p* = 0.0002, Fig. 4H; *t* = 0.741, *p* = 0.470, Fig. 4I). The activities of FDH, ALDH2 and SSAO were not changed in the VTA and PFC (*t* = 0.326, *p* = 0.749, Fig. 4J; *t* = 0.233, *p* = 0.819, Fig. 4K; *t* = 0.435, *p* = 0.670, Fig. 4L; *t* = 0.477, *p* = 0.640, Fig. 4M; *t* = 0.284, *p* = 0.780, Fig. S1C; *t* = 0.452, *p* = 0.658, Fig. S1D). Notably, there was a markedly increase in FA levels in the VTA and PFC in the TIQ-model mice than control (*t* = 3.295, *p* = 0.005, Fig. 4N; *t* = 3.107, *p* = 0.007, Fig. 4O).

**Fig. 5:**
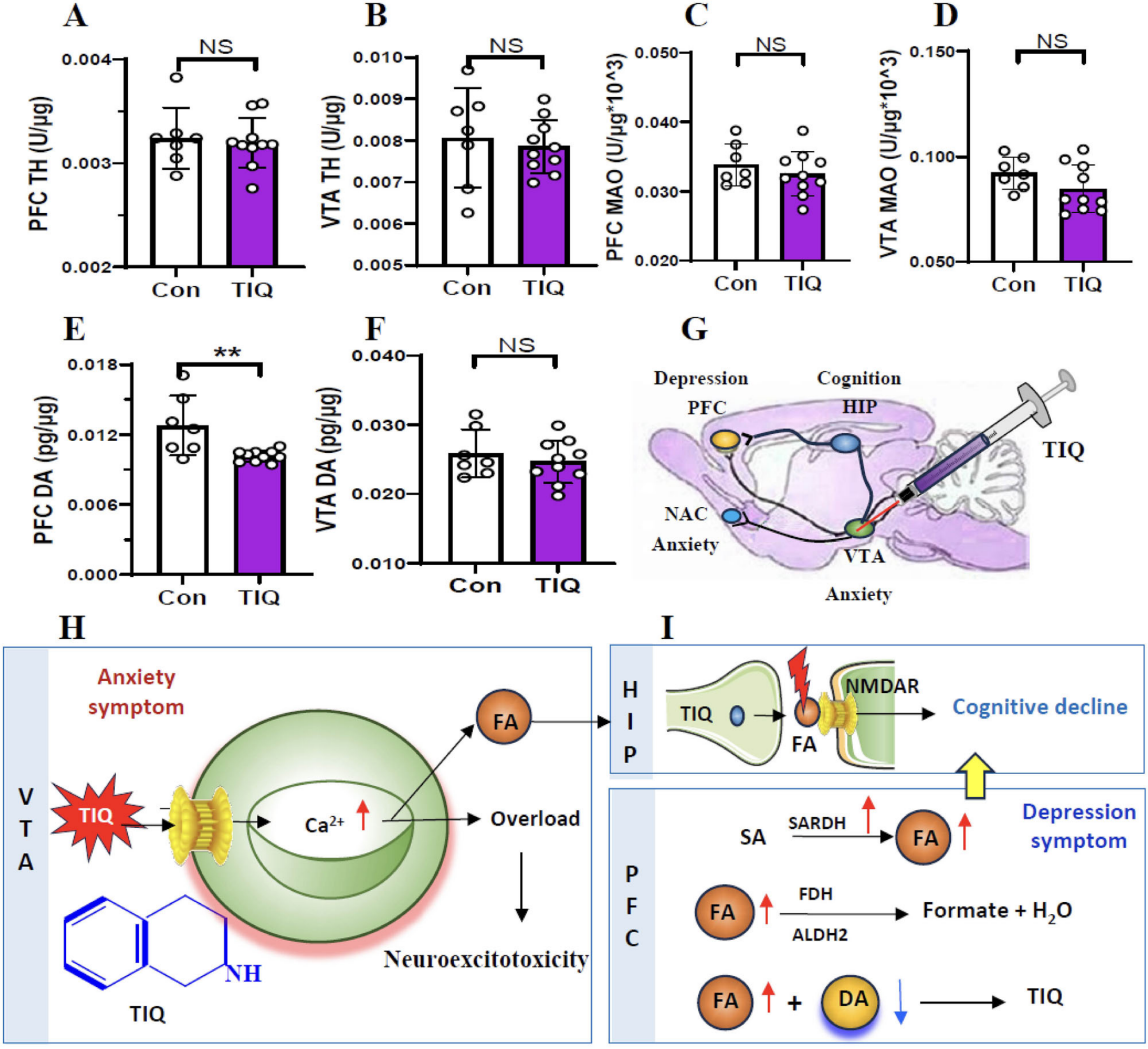
Changes in the metabolic levels of DA in the TIQ-model mice. **A**, **B** TH activity in the PFC and VTA detected by ELISA kits. TH tyrosine dehydrogenase, PFC prefrontal cortex, VTA ventral tegmental area, ELISA enzyme-linked immunosorbent assay. **C**, **D** MAO activity in the PFC and VTA detected by ELISA kits. MAO monoamine oxygenase. **E**, **F** DA levels in the PFC and VTA quantified by ELISA kits. DA dopamine. **G** Location and injection of TIQ. **H** Anxiety associated with high levels of TIQ in the VTA. **I** Cognitive decline by FA-blocked NMDAR and depression accompanied by low levels of DA in the PFC. *p < 0.05; **p < 0.01; NS no statistical significance.

Next, we examined the metabolic levels of DA in the VTA and PFC in the mice. We found that the activities of both TH and MAO were not changed in the VTA and PFC in the TIQ-mice than control (*t* = 0.334, *p* = 0.743, Fig. 5A; *t* = 0.470, *p* = 0.645, Fig. 5B; *t* = 0.866, *p* = 0.400, Fig. 5C; *t* = 1.541, *p* = 0.114, Fig. 5D). Compared with control, the DA levels were decreased in the PFC but not in the VTA in the TIQ-mice (*t* = 3.184, *p* = 0.006, Fig. 5E; *t* = 0.755, *p* = 0.462, Fig. 5F). Above data indicate that TIQ-stimulated VTA leads to neuroexcitotoxicity in the VTA^35,36^, which contributes to anxiety onset (Fig. 5G, H). However, excessive FA can inactivate DA; low levels of DA in the PFC cause cognitive decline and depression occurrence (Fig. 5I).

### Elevated VTA TIQ levels and reduced PFC DA levels in the FA-model mice

**Fig. 6:**
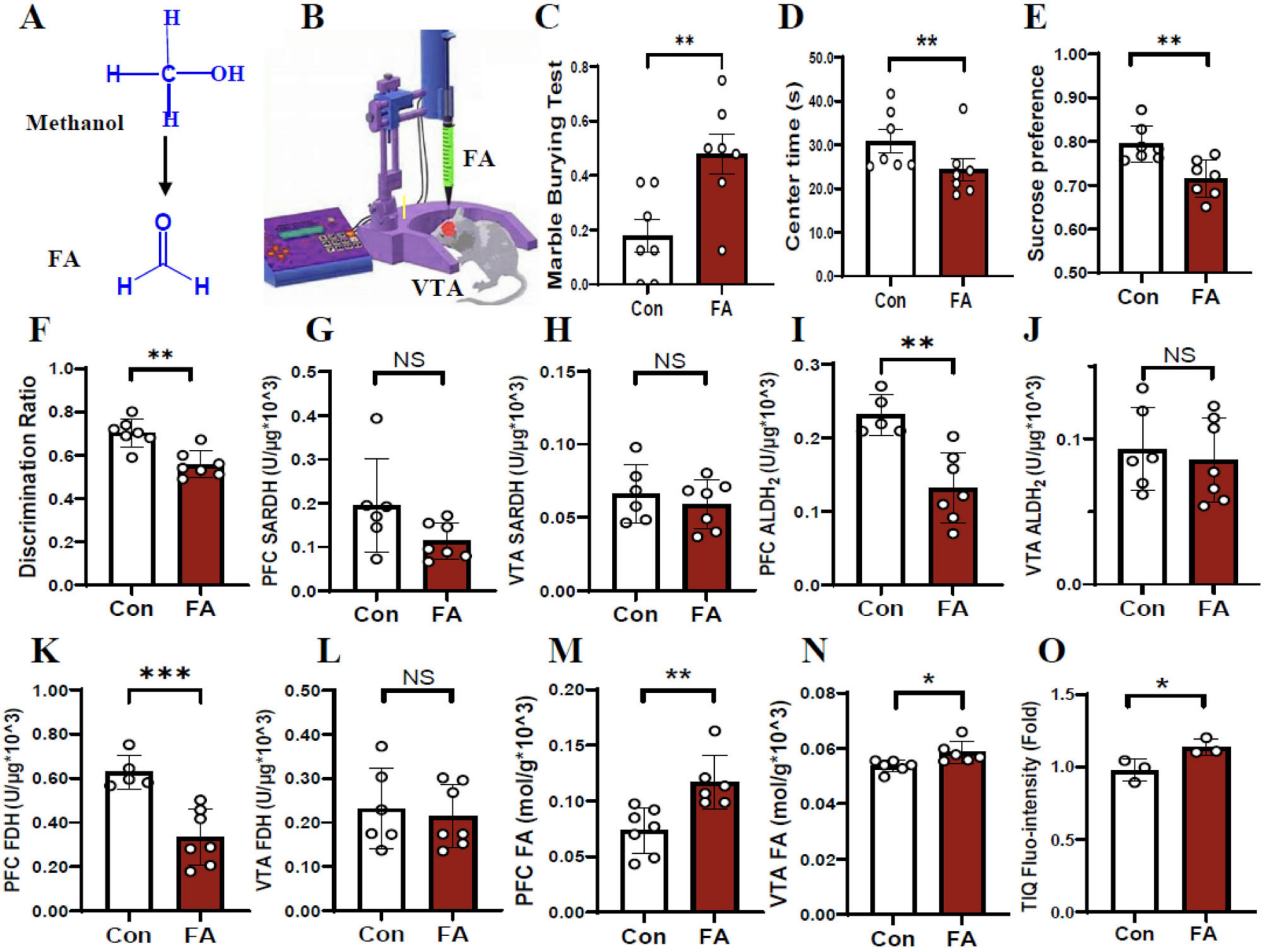
Changes in behaviors and the metabolic levels of FA in the FA-model mice. **A, B** Microinfusion of FA into the VTA in mice. FA formaldehyde, VTA ventral tegmental area. **C**, **D** Anxiety-like behaviors assessed by the marble burying test (MBT) and open-field test (OFT), respectively. **E** Depression-lie behaviors evaluated by the tail suspension test (TST) and sucrose preference test (SPT), respectively. **F** Short-term memory assessed by Y-maze test. **G**, **H** SARDH activity in the PFC and VTA detected by ELISA kits. ELISA enzyme-linked immunosorbent assay, PFC prefrontal cortex, SARDH sarcosine dehydrogenase. **I**, **J** ALDH2 activity in the PFC and VTA detected by ELISA kits. ALDH2 aldehyde dehydrogenase 2. **K**, **L** FDH activity in the PFC and VTA detected by ELISA kits. FDH formaldehyde dehydrogenase. **M**, **N** FA levels in the PFC and VTA detected by NaFA probe. FA formaldehyde. **O** TIQ levels detected by HPLC. HPLC high-performance liquid chromatography. *p < 0.05; **p < 0.01; NS no statistical significance.

Since FA is the critical factor of TIQ formation and DA inactivation^25^, we explored the effects of excessive FA in the VTA on behaviors in mice. Brain stereotactic injection of FA was applied into VTA of mice (Fig. 6A, B), and then behaviors including anxiety, depression and memory were assessed. We found that the FA-mice showed anxiety-like behaviors, because they had an increase in the MBT but a decrease in the movement time in the central zone in the OFT than control (*t* = 3.146, *p* = 0.008, Fig. 6C; *t* = 4.047, *p* = 0.007, Fig. 6D). The FA-mice also exhibited depression-like behaviors associated with a decrease in the preference in the SPT than control (*t* = 3.451, *p* = 0.005, Fig. 6E), and memory deficits with lower percentage of alternating behaviors in the YMT (*t* = 4.340, *p* = 0.001, Fig. 6F).

**Fig. 7:**
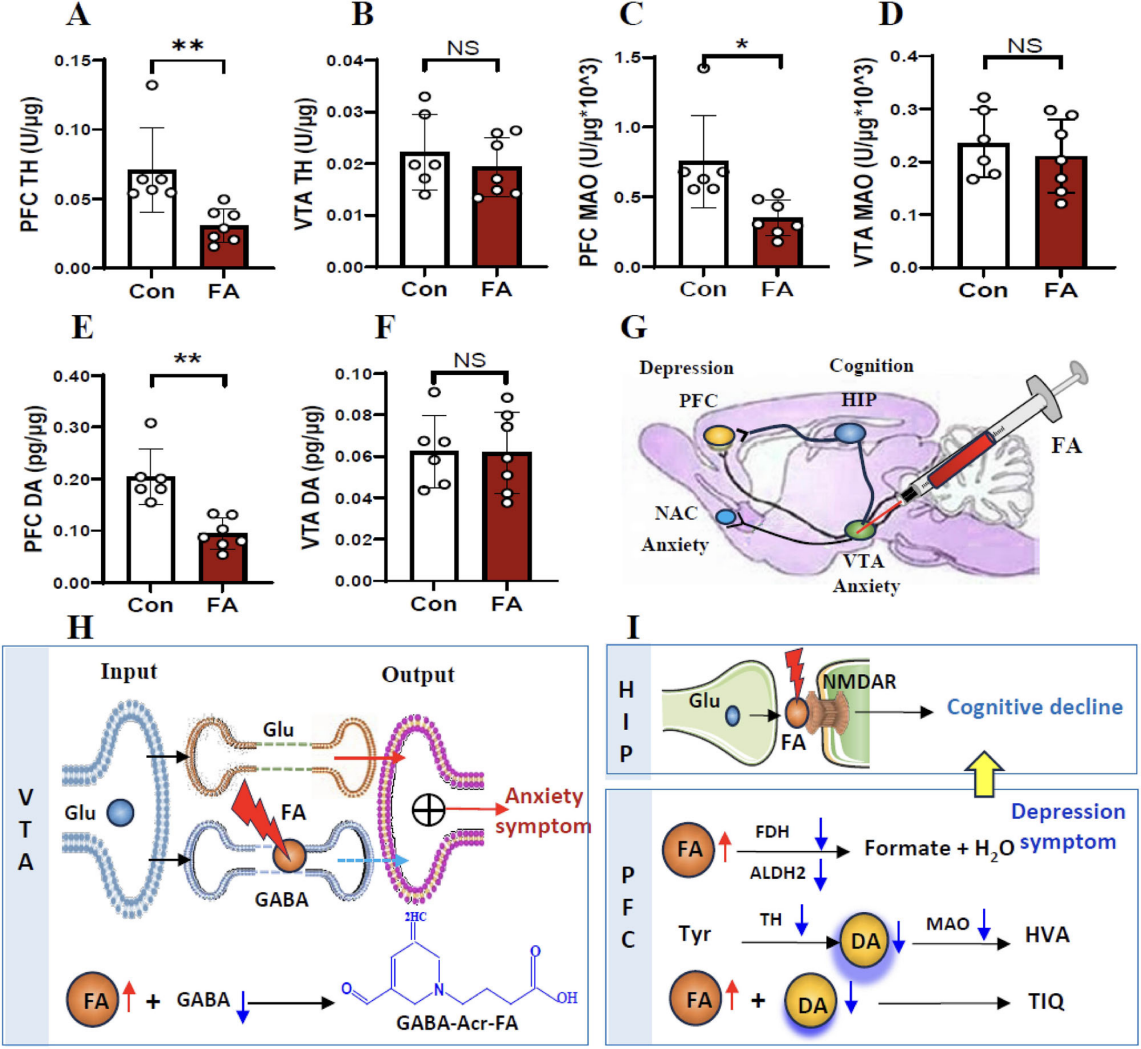
Changes in the metabolic levels of DA in the FA-model mice. **A**, **B** TH activity in the PFC and VTA detected by ELISA kits. TH tyrosine dehydrogenase, PFC prefrontal cortex, VTA ventral tegmental area, ELISA enzyme-linked immunosorbent assay. **C**, **D** MAO activity in the PFC and VTA detected by ELISA kits. MAO monoamine oxygenase. **E**, **F** DA levels in the PFC and VTA quantified by ELISA kits. DA dopamine. **G** Location and injection of FA. **H** Anxiety associated with low levels of GABA in the VTA. **I** Cognitive decline by FA-blocked NMDAR and depression accompanied by low levels of DA in the PFC. *p < 0.05; **p < 0.01; NS no statistical significance.

Next, we found that the activities of SARDH and SSAO was not changed in the VTA and PFC in the FA-mice than control (*t* = 1.833, *p* = 0.094, Fig. 6G; *t* = 0.718, *p* = 0.488, Fig. 6H; *t* = 1.951, *p* = 0.080, Fig. S1E; *t* = 0.619, *p* = 0.549, Fig. S1F). However, the activities of ALDH2 and FDH were decreased in the PFC but not in the VTA in the FA-mice (*t* = 4.185, *p* = 0.002, Fig. 6I; *t* = 0.455, *p* = 0.658, Fig. 6J; *t* = 4.632, *p* = 0.0009, Fig. 6K; *t* = 0.387, *p* = 0.706, Fig. 6L). Notably, there was an increase in FA levels in both VTA and PFC and elevation in TIQ levels in the VTA in the FA-model mice than control (*t* = 3.506, *p* = 0.005, Fig. 6M; *t* = 2.746, *p* = 0.021, Fig. 6N; *t* = 2.900, *p* = 0.044, Fig. 6O). Furthermore, we found that the activities of both TH and MAO were decreased in the PFC but not in the VTA in the FA-mice than control (*t* = 3.224, *p* = 0.008, Fig. 7A; *t* = 0.787, *p* = 0.448, Fig. 7B; *t* = 2.969, *p* = 0.013, Fig. 7C; *t* = 0.641, *p* = 0.534, Fig. 7D). Compared with control, the DA levels were decreased in the PFC but not in the VTA in the FA-mice (*t* = 4.714, *p* = 0.0006, Fig. 7E; *t* = 0.053, *p* = 0.958, Fig. 7F).

### Reduced PFC DA levels in the MK801-model mice

**Fig. 8:**
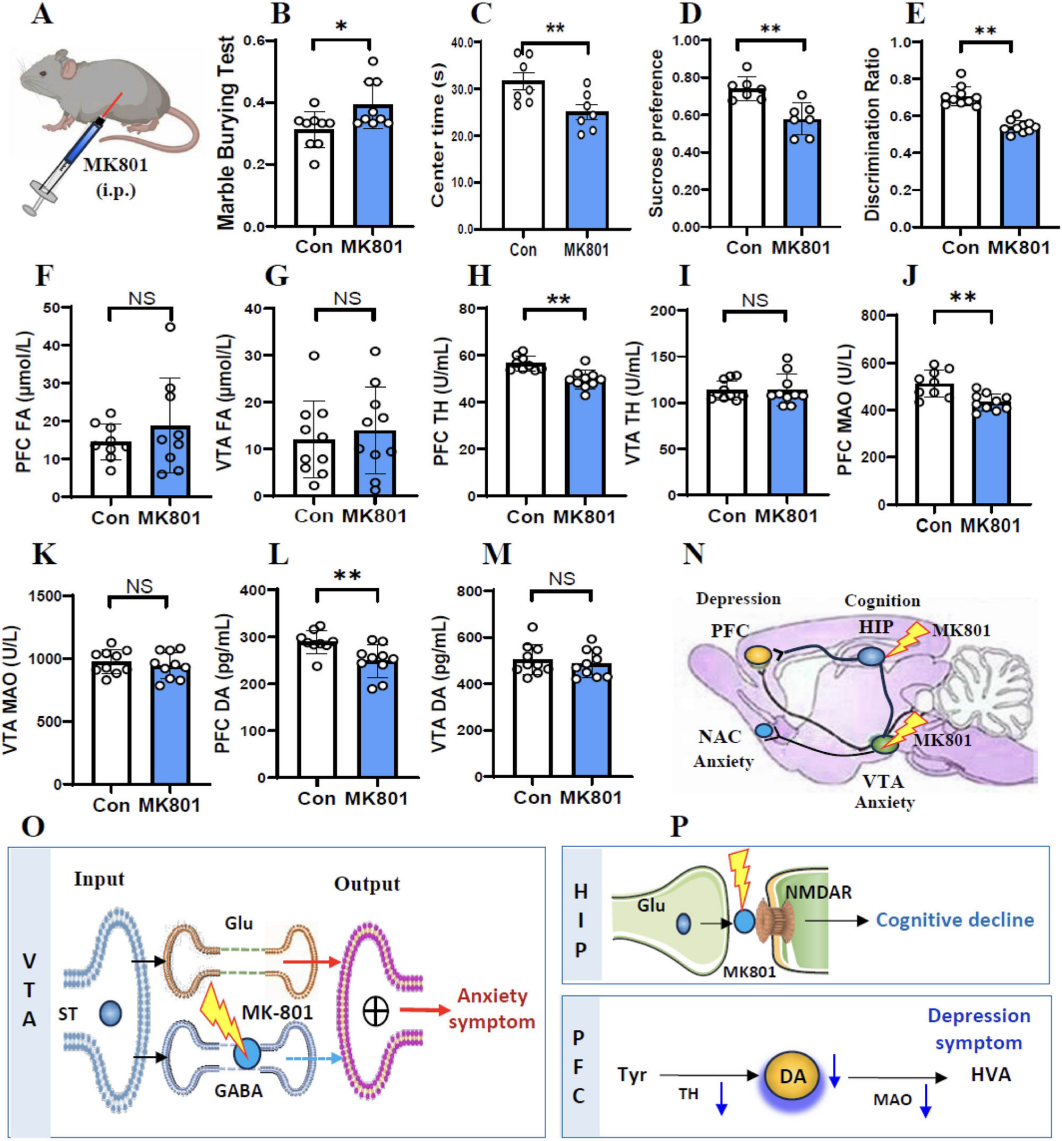
Changes in behaviors and the metabolic levels of DA and FA in the MK801-model mice. **A** Microinfusion of MK801 into the VTA in mice. VTA ventral tegmental area. **B**, **C** Anxiety-like behaviors assessed by the marble burying test (MBT) and open-field test (OFT), respectively. **D** Depression-lie behaviors evaluated by the tail suspension test (TST) and sucrose preference test (SPT), respectively. **E** Short-term memory assessed by Y-maze test. **F**, **G** FA levels in the PFC and VTA detected by NaFA probe. FA formaldehyde. **H**, **I** TH activity in the PFC and VTA detected by ELISA kits. TH tyrosine dehydrogenase, PFC prefrontal cortex, VTA ventral tegmental area, ELISA enzyme-linked immunosorbent assay. **J**, **K** MAO activity in the PFC and VTA detected by ELISA kits. MAO monoamine oxygenase. **L**, **M** DA levels in the PFC and VTA quantified by ELISA kits. DA dopamine. N Location and injection of MK801. **O** Anxiety associated with low activity of GABA neurons in the VTA. **P** Cognitive decline by FA-blocked NMDAR and depression accompanied by low levels of DA in the PFC. *p < 0.05; **p < 0.01; NS no statistical significance.

To investigate the common mechanisms in the FA-injected model and MK801-injected model mice, the wild-type mice were intraperitoneally injected with MK801 (Fig. 8A). We found that the MK801-mice showed anxiety-like behaviors, because they had an increase in the MBT but a decrease in the movement time in the central zone in the OFT than control (*t* = 2.486, *p* = 0.024, Fig. 8B; *t* = 4.505, *p* = 0.004, Fig. 8C). The FA-mice also exhibited depression-like behaviors associated with a decrease in the preference in the SPT than control (*t* = 4.009, *p* = 0.002, Fig. 8D), and memory deficits with lower percentage of alternating behaviors in the YMT (*t* = 8.101, *p* < 0.0001, Fig. 8E).

Next, we found that the activities of SARDH, ALDH2 and FDH were not changed in the VTA and PFC in the MK801-mice than control (*t* = 1.988, *p* = 0.063, Fig. S2A; *t* = 0.892, *p* = 0.384, Fig. S2B; *t* = 1.353, *p* = 0.194, Fig. S2C; *t* = 0.046, *p* = 0.964, Fig. S2D; *t* = 0.332, *p* = 0.751, Fig. S2E; *t* = 2.130, *p* = 0.065, Fig. S2F). However, the activity of SSAO were decreased in the PFC but not in the VTA in the MK801-mice (*t* = 3.459, *p* = 0.003, Fig. S2G; *t* = 1.290, *p* = 0.004, Fig. S2H). Notably, there was no changes in FA levels in both VTA and PFC in the MK801-model mice than control (*t* = 0.928, *p* = 0.367, Fig. 8F; *t* = 0.490, *p* = 0.630, Fig. 8G). Furthermore, we found that the activities of both TH and MAO were decreased in the PFC but not in the VTA in the MK801-mice than control (*t* = 4.250, *p* = 0.0005, Fig. 8H; *t* = 0.070, *p* = 0.945, Fig. 8I; *t* = 3.665, *p* = 0.002, Fig. 8J; *t* = 0.695, *p* = 0.496, Fig. 8K). Compared with control, the DA levels were decreased in the PFC but not in the VTA in the MK801-mice (*t* = 2.946, *p* = 0.009, Fig. 8L; *t* = 0.575, *p* = 0.573, Fig. 8M).

## Discussion

In this study, we established an innovative murine model of schizophrenia-like changes through the microinjection of FA into the VTA (Fig. S3). First, we found that double electrode stimulation of VTA elicited a release of DA in the VTA but a decrease in DA levels in the PFC, and TIQ formed between FA and DA in the VTA. Unexpectedly, microinjection of FA or TIQ into the VTA as well as intraperitoneal injection of MK801, all leads to anxiety-like behaviors^38^. Because the imbalance between Glu- and GABAergic neurons induces depression^18,19^ (Fig. 7G, H), depression-like behaviors decline for low levels of DA in the PFC (Fig. 8N–P), and memory by blocking NMDAR in mice. These findings suggest that stress-derived FA is an endogenous inducer of schizophrenia-like changes and provide evidence for the hypothesis that high levels of DA in the midbrain limbic leads to positive symptoms, and low dopamine activity in the midbrain cortex causes negative and cognitive symptoms^9,15^.

FA is present in all cells and is highly reactive, chemically reacting with a variety of neurotransmitters in the body^39^. Both gaseous and liquid FA directly promote depression, and FA induces oxidative stress and promotes inflammation^40^, Inflammatory cytokines can have an effect on the metabolism of monoamine neurotransmitters^41^. Previous studies have proposed that FA reduces DA and 5-HT production, which is strongly associated with the development of depression^22,42,43^. Notably, TIQ can be formed from DA and FA^25^. TIQ can cross the blood-brain barrier and affect brain functions^27^, which has neurotoxic effects and affect behaviors^44–46^.

It is noteworthy that although the changes in FA concentrations in the VTA regions are not all consistent in these four kinds of animal models of schizophrenia-like changes, endogenous FA may play a common role in the toxicity of DA-nergic neurons. In the ES-induced model mice, the neurons in the VTA contain high concentrations of DA. ES induces DA release (Fig. 2F) and FA generation in the VTA; however, FA had a spontaneous reaction with DA to form TIQ and resulted in a decline in FA levels (Fig. 1O). High levels of TIQ led to neuron death^24,25^. In the TIQ-injected model mice, TIQ-induced intracellular Ca^2+^ influx leads to a marked increase in the FA concentrations in the VTA (Fig. 4O), which directly damaged DA-nergic neurons. Consequently, FA induced a slight decline in DA levels in the VTA for the spontaneous chemical reaction between these two compounds (Fig. 5F). In the FA-injected model mice, injection of aqueous FA increased FA concentrations in the VTA (Fig. 6N) and directly induced the toxicity of DA-nergic neurons. FA can directly inactivate GABA^47,48^. Hence, low levels of GABA in the VTA and low levels of DA in the PFC lead to anxiety and depression in schizophrenia-like changes (Fig. 7I)

In the MK801-injected model mice, FA-inhibited NMDA-R contributed to schizophrenia-like changes onset. The NMDAR hypofunction hypothesis of schizophrenia stems from the finding that non-competitive NMDAR antagonists like PCP and ketamine produce behaviors similar to the positive, negative, and cognitive symptoms of schizophrenia in humans^16^. Previous study has revealed that external stress-derived FA can block NMDAR in the hippocampus, which directly impairs memory^23^. Hence, microinjection of FA into VTA caused memory decline in mice in this study (Fig. 8O, P). In addition, low activity of GABAergic neurons and high activity of Glu neurons in the VTA^18,19^, lead to anxiety^38^. However, FA can inactivate DA, and low levels of DA in the PFC cause depression and cognitive impairments in schizophrenia-like changes.

Remarkably, the injection of FA into the VTA induced extensive pathophysiological alterations not only in the VTA but also in the PFC and other brain regions. This model successfully simulated the positive, negative, and cognitive symptoms associated with clinical schizophrenia, distinguishing it from previous animal models that typically exhibited only a single symptom. Clinically, the exogenous intake of methanol, a precursor in FA metabolism, and aspartame, an FA releaser, has been associated with hallucinations, anxiety, and other symptoms characteristic of schizophrenia^49^. In addition, FA administration resulted in a reduction of DA levels in the PFC and a downregulation of the activity of related metabolic enzymes such as TH and MAO. This finding aligns with the observed downregulation of MAO activity in the blood of patients with schizophrenia and supports the negative symptom hypothesis of dopamine deficiency^50,51^.

The current study is subject to certain limitations. First, the cause of the decline in DA levels in the PFC following TIQ injection was not identified in this study. However, this observation aligns with findings from previous research and may elucidate the emergence of negative symptoms and cognitive impairments. A potential explanation is that the direct administration of TIQ induces the release of calcium ions (Ca^2+^), which subsequently FA has chemical reaction with DA, leading to a reduction of DA in the PFC. Second, we did not differentiate whether the observed memory impairment was attributable to excessive FA levels resulting from suppressed NMDAR activity in the hippocampus, or due to reduced excitability in the PFC for DA deficiency. It is possible that both mechanisms contribute to memory deficits. Third, although external stimulation or stress is a well-established inducer of schizophrenia, this study did not establish a direct relationship between the intensity of stimulation or the extent of neuronal excitation-derived FA and the onset of schizophrenia. Fourth, although this study successfully established a novel animal model of schizophrenia-like change through the microinfusion of FA into the VTA, the duration for which this model sustains the three characteristic symptoms remains uncertain. The following issues require further investigation: (1) The comparative efficacy of intraperitoneal injection versus microinfusion of FA into the hippocampus at pathological concentrations in inducing schizophrenia-like symptoms in mice. (2) The potential for varying doses of FA to elicit both positive and negative symptoms in these FA-injected model mice. (3) The suitability of this FA-injected model as a pharmacological tool for the development of novel therapeutic agents for schizophrenia. Finally, our study exclusively utilized male mice to develop the mouse model of schizophrenia-like behaviors. While the clinical prevalence of schizophrenia is notably higher in males compared to females, female patients exhibit distinct disease trajectories and pathophysiological characteristics, including a later onset and the potential protective influence of estrogen^52^. Thus, further exploration into the female mouse model of schizophrenia is warranted.

In summary, our findings suggest that stress-induced FA may function as an endogenous trigger for schizophrenia. This is evidenced by the injection of FA into the VTA, which led to increased activity in VTA-associated positive behaviors and concurrently decreased activity in PFC-associated negative behaviors in mice, thereby replicating the symptomatic manifestations observed in clinical schizophrenia. These insights are advantageous for elucidating the pathological mechanisms underlying schizophrenia and for accelerating the development of novel therapeutic interventions.

## Supplementary information

Data Set 3

## Data Availability

The data analyzed in this study can be available in this published article and its supplementary information files.
