# Supplementary material for: External stress, formaldehyde, and schizophrenia: a new mouse model for mental illness research
Source: Schizophrenia (Heidelb). 2025 Mar 26;11(1):50. doi: 10.1038/s41537-025-00603-3 (PMC11947252; doi:10.1038/s41537-025-00603-3)
Supplement: Supplementary file 1 — Data Set 3 [file 41537_2025_603_MOESM1_ESM.pdf]

Supplementary Figure 1

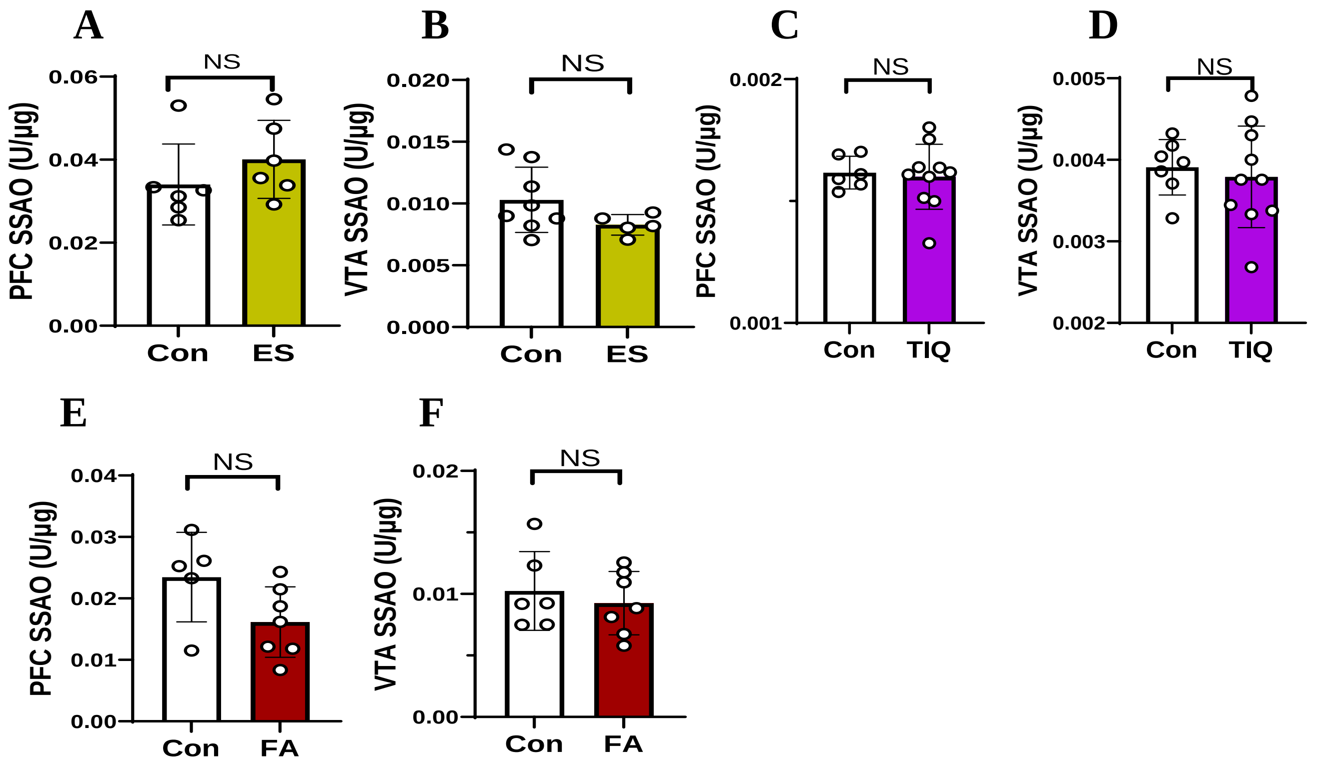

Figure S1. Changes in the SSAO activity in the VTA and PFC in the different model mice quantified by ELISA kits. SSAO: semicarbazide-sensitive amine oxidase. PFC: prefrontal cortex. VTA: ventral tegmental area. ELISA: enzyme-linked immunosorbent assay. NS: no statistical significance.

Supplementary Figure 2

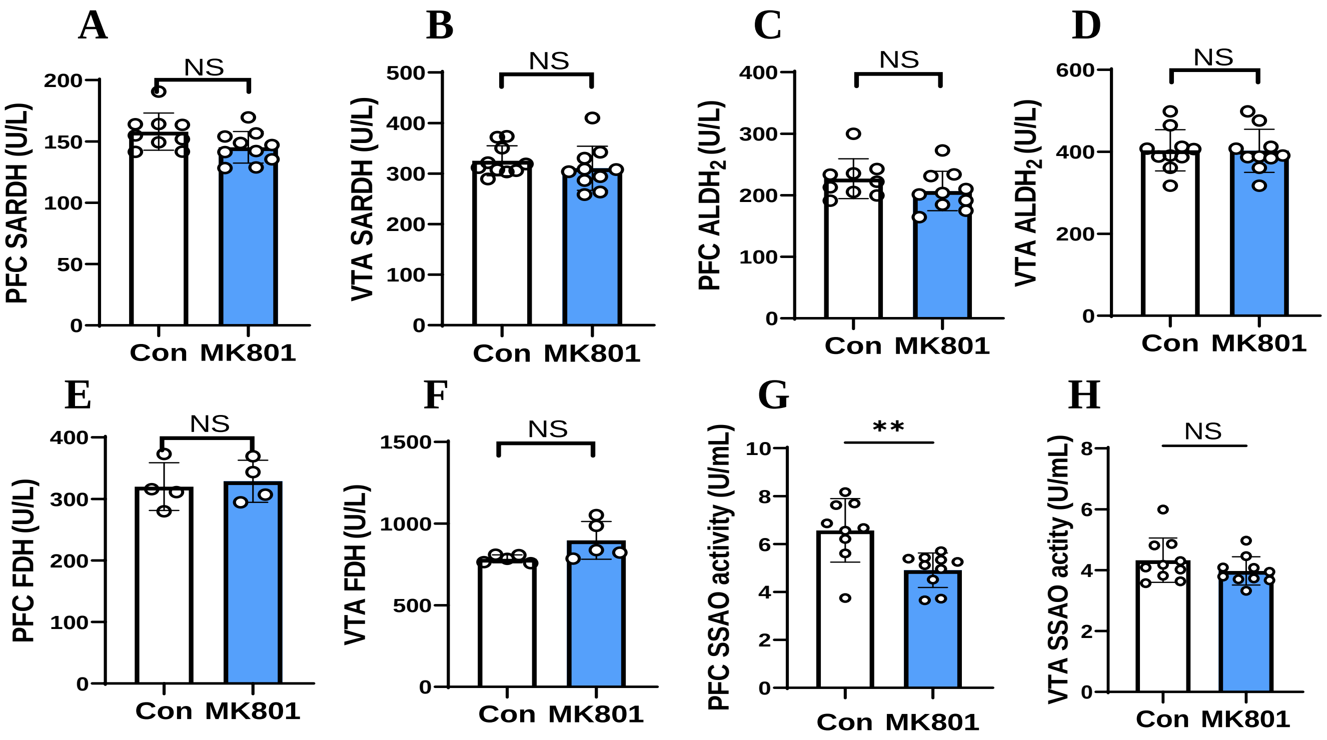

Figure S2. Changes in the activities of SARDH, ALDH2 and FDH in the VTA and PFC in the MK801-model mice quantified by ELISA kits. ALDH2: aldehyde dehydrogenase 2. FDH: formaldehyde dehydrogenase. SSAO: semicarbazide-sensitive amine oxidase. PFC: prefrontal cortex. VTA: ventral tegmental area. ELISA: enzyme-linked immunosorbent assay. ** $p<0.01$; NS: no statistical significance.

Supplementary Figure 3

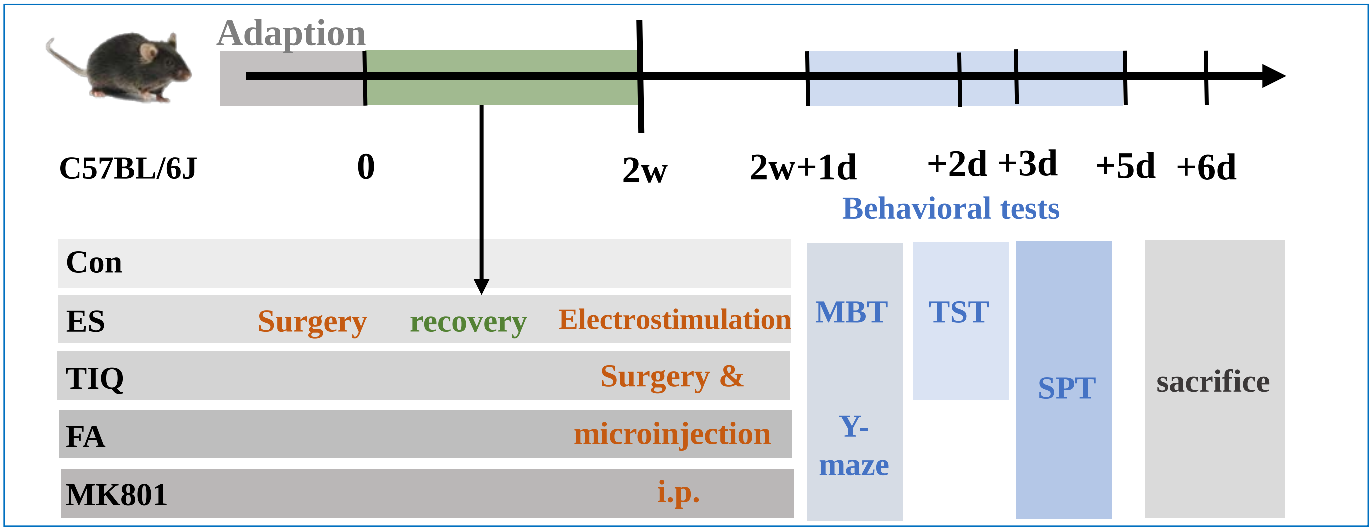

Figure S3. Experimental flow charts of the four kinds of model mice. The time point from adaptation to stimulation, behavior assessments, and biochemical tests after sacrifice. d: day; i.p.: intraperitoneal injection; MBT: marble burying test; OFT: open-field test; TST: tail suspension test; SPT: sucrose preference test.
